# The role of gender on malaria preventive behaviour among rural households in Kenya

**DOI:** 10.1186/s12936-015-1039-y

**Published:** 2016-01-07

**Authors:** Gracious M. Diiro, Hippolyte D. Affognon, Beatrice W. Muriithi, Sarah Kingori Wanja, Charles Mbogo, Clifford Mutero

**Affiliations:** International Center of Insect Physiology and Ecology (ICIPE), Nairobi, Kenya; Makerere University , Kampala, Uganda; Kenya Medical Research Institute (KEMRI), Kilifi, Kenya; UP Centre for Sustainable Malaria Control, University of Pretoria, Pretoria, South Africa; International Crops Research Institute for the Semi-Arid Tropics (ICRISAT), Bamako, Mali

**Keywords:** Malaria preventive behaviour, Count models, Gender, Kenya

## Abstract

**Background:**

Malaria remains a major health and development challenge in the sub-Saharan African economies including Kenya, yet it can be prevented. Technologies to prevent malaria are available but are not universally adopted by male- and female-headed households. The study thus, examined the role of gender in malaria prevention, examining adoption behaviour between male- and female-headed households in Kenya.

**Methods:**

The study uses a recent baseline cross-section survey data collected from 2718 households in parts of western and eastern Kenya. Two separate models were estimated for male- and female-headed households to determine if the drivers of adoption differ between the two categories of households.

**Results:**

The findings from the study show that: access to public health information, residing in villages with higher experience in malaria prevention, knowledge on the cause and transmission of malaria significantly increase the number of practices adopted in both male- and female-headed households. On the other hand, formal education of the household head and livestock units owned exhibited a positive and significant effect on adoption among male-headed households, but no effect among female-headed households.

**Conclusions:**

The findings from thus study suggest that universal policy tools can be used to promote uptake of integrated malaria prevention practices, for female- and male-headed households.

## Background

Malaria remains a major public health concern across many sub-Saharan African (SSA) economies [1, 2]. In Kenya, malaria accounts for 19 % of hospital admissions, 3–5 % of inpatient deaths and between 30–50 % of outpatient cases [3], and an estimated 74 % of the population is at risk of getting the disease [4]. High malaria burden hampers economic growth through its adverse effects on the agricultural sector [5–7] the dominant sector for Kenya [8]. Agriculture contributes a third of the regional gross national product (GNP) and employs at least two-thirds of the labor force [8]. At the household level, malaria reduces labour productivity of the members, increases health expenditure and reduces the capacity of households to accumulate assets [5, 7, 9].

The Government of Kenya, in collaboration with other partners have, over the years, implemented the malaria control programme [3]. The Kenya National Malaria Control Programme (KNMCP, 2009–2017) aims at increasing access and utilization of interventions for malaria prevention and control. The priority intervention areas for the KNMCP (2009–2017) include promoting large-scale use of long-lasting insecticide-treated nets (LLINs), indoor residual spraying (IRS), environmental management to prevent mosquito breeding, prevention of malaria in pregnancy through insecticide-treated nets (ITNs) and intermittent preventive treatment of malaria in pregnancy (IPTp) and improved appropriate case management including parasite-based diagnosis and treatment with artemisinin-based combination therapy (ACT) [3]. Despite the effort devoted to prevent and control malaria in Kenya, prevalence of the disease remains high, particularly in the areas around Lake Victoria where the parasite rate is reportedly around 38 % [10]. In addition, use of the technologies for preventing malaria is still low although they are widely available. Recent results from the 2008–2009 Kenya Demographic and Health Survey (KDHS) show that just about half of the sampled households owned an ITN in 2009, and only 32 % had more than one ITN. The statistics further show that only 49 % of pregnant women slept under an ITN, and 42 % of women took any anti-malarial drugs during pregnancy in 2008–09. Further, only 47 % of children under-five slept under an ITN in 2008–2009.

The literature has put forth several explanations for low utilization of malaria control interventions in Africa. Research suggests that high costs, long distance to health facilities, limited knowledge of malaria or and practices are some of the main deterrents to uptake of malaria prevention and control interventions in Africa [11–14]. Studies also show that formal education is important for appropriate prevention and treatment strategies [11, 15–18]. However, others such as [19, 20] find a significantly higher rate of ITN use among less educated pregnant women in Uganda, and Nigeria—a finding they attribute to increased perceived vulnerability to malaria in poorer households. In particular, [20] finds that women without formal education, in Nigeria, were 1.75 times more likely to sleep under an ITN than those with post secondary education. Economic constraints and inequities in household resources also affect access to ITNs and are an important factor in malaria prevention and control [21, 22]. For instance [21] reports that women in Benin, who earn an income and had control over this income, were much more likely than men to purchase an ITN for their household.

However, the extant empirical literature on malaria preventive behaviour has two main gaps. The first is that past studies mainly analyse adoption of individual technology, ignoring multiple interventions a household may adopt to prevent and control malaria. Indeed, most of the existing studies such as those discussed above focus on treatment-seeking behaviour and prophylaxis, leaving out interventions such as environmental management and source reduction that control the vector population. Given that many technologies with different attributes are available, it is important to understand why some households adopt none or fewer technologies than others. The second limitation is that existing studies on malaria preventive behaviour, particularly those conducted in Kenya [13, 16, 22], have not addressed the potential role of gender in malaria control. In many parts of the world including Kenya, women play the primary role of care giving to other members in the household, including leading the majority of health care seeking for the rest of the family members [23]. However, men still dominate decision-making on health and economic issues in households, which is likely to affect success of health care interventions such as malaria control and prevention programmes. Understanding the role of gender in adoption of malaria prevention and control in households will be important in improving the coverage and effectiveness of malaria control and prevention strategies in the country.

The present study contributes to existing literature on malaria prevention and control in Kenya in the following ways: The study uses a more recent data-set (of 2718 households) to analyse adoption of interventions to prevent malaria vectors. Unlike most of the previous studies cited above, the current study analyses household adoption of complementary interventions for malaria prevention and control, with a focus on the effect of gender. That is, separate adoption models for male- and female-headed households were estimated to determine if the drivers of adoption differ between the two categories of households.

### Economic model

In this study health-seeking behaviour for malaria prevention in Kenya was conceptualized using a random utility framework [24, 25]. In that respect, the study assumes that households make rational decisions regarding the disease prevention, and thus choose practices that maximize their expected net benefits. The random household utility (Uik) derived from choice of practice k is presented in Eq. (1):

1 $${\text{U}}_{\text{ik}} = {\text{V}}_{\text{ik}} + \varepsilon_{\text{ik}} ,\quad {\text{ for i}} = 1, \ldots ,{\text{n}}$$

where V_ik_ is deterministic component, ε_ik_ is the error term (representing unobserved attributes that influence malaria preventive behaviour, heterogeneity in tastes and measurement errors). Individual household utility (V_ik_) is assumed to be a linear function of the attributes of the technology (Sk) that vary across choices, and the socio-economic characteristics of the household (wi) that are common to all choices, represented as (2):

2 $${\text{V}}_{\text{ik}} { = }\,\beta {\text{w}}_{\text{ik}} \,{ + }\,\varphi_{\text{k}} {\text{S}}_{\text{ik}} { = }\,{\text{x}}_{\text{ik}} \theta ,\quad {\text{for}}\,{\text{i}}\,{ = }\, 1 ,\ldots ,{\text{n}}$$

where x_ik_ is the arithmetic combination representing the covariates s_ik_ and wik; $\theta \equiv \{ \beta ,\,\,\varphi k\}$ is the vector of parameters. The study assumes that households in Kenya make rational decisions on malaria prevention and, therefore, decide to adopt a given technology (health practice) k if its utility is higher than for all other choices; that is, $V_{ik} \text{ + }\varepsilon_{ik} \text{ > }U_{ij} \text{ + }\varepsilon_{ij}$ for all ${\text{k}} \ne {\text{j}}$. The response model of use of malaria preventive practices k over j is thus specified in Eq. (3):

3 $${\text{y} = \text{g}}\left( {x^{\prime}_{\text{i}} \theta + \varepsilon_{\text{ik}} - x^{\prime}_{\text{ij}} \theta - \varepsilon_{\text{ij}} { > 0}} \right) {= \text{ g}}\left( {x^{\prime}_{\text{i}} + \mu_{\text{i}} } \right) , {\text{ for i = 1,}} \ldots , {\text{n}}$$

where $\mu_{\text{i}} = \, \varepsilon_{\text{ik}} - \varepsilon_{\text{ij}}$ is random error term with zero mean, g is some distribution function of µ_i_.

### Empirical methods

The empirical analysis in this study considers seven integrated practices for prevention and control of malaria that are available to the farmer households. These include use of indoor spray, purchased repellents, traditional repellents (herbs), screening (windows and doors), proper drainage around homes, bush clearing, and proper disposal of empty containers and other trash that would otherwise provide conducive breeding sites for mosquitoes. Some of these practices work together but can be used individually. The number adopted by a given household depends on the perceived need and capacity of the household. A household may not adopt any of the practices or may adopt some or all the practices recommended and available for mosquito prevention and control. Use of bed nets was excluded in the dependent variable to avoid introducing bias the variable, as bed nets are commonly used in households. For example about 96 % of the female-headed households and 94 % of their male-headed counterparts in the study area own bed nets. Bed nets are supplied to the households free of charge by the Government of Kenya and development partners in health sector; through the roll back malaria strategy.

Extant literature on technology adoption mainly treats adoption as categorically ordered variables, undertaking values such as “none, low, average, high and total”. Others studies have analysed adoption of individual technologies, by estimating individual binary choice models. This may be inefficient because agents make simultaneous choices. Some studies [26], transform the count ordered variable for adoption into a binomial variable by, for example, assigning a value of one when adoption was high or total and zero otherwise. Most of these approaches may introduce measurement errors in the dependent variable.

Further, a stepwise or partial adoption process may not be measured by a dichotomous dependent variable. The present study therefore applies count models to analyze the adoption of malaria prevention interventions among subsistence farmer households in Kenya. The study specifically focuses on the number of prevention technologies a household has adopted. In this case, the dependent variable is a count, defined as the probability that a household chooses a number of malaria prevention practice as specified in Eq. (4):

4 $${\text{pr}}\left( {{\text{choosing}}\,{\text{k} = \text{q}}_{\text{k}} } \right) = \frac{{\exp \left( {{ - }\lambda_{k} } \right)\lambda_{k}^{{q_{k} }} }}{{q_{k} \text{!}}}$$

where q_k_ is the number of malaria prevention practices chosen by household i; λ_k_ is the average number of technologies chosen, that is;

$$\lambda_{\text{k}} = {\text{E}}\left( {{\text{q}}_{\text{k}} |x} \right) = { \exp }\left( {x^{\prime}_{\text{i}} \theta } \right)$$

Practically, $$\overset{\lower0.5em\hbox{$\smash{\scriptscriptstyle\frown}$}}{\theta }$$ is chosen by maximizing the likelihood function (Eq. 5)

5 $$L\left( \theta \right) = \sum\limits_{i = 1}^{n} {\{ \exp \left( {x^{\prime}_{i} \theta } \right) + q_{k} x^{\prime}} \theta - \ln q_{k} {!\} }$$

where, L is the log likelihood function. Equation 5 was implemented using a simple poisson regression model. A test for over dispersion of the data was performed to determine the suitability of the Poisson model against the Negative Binomial. The null that the data is over-dispersed was rejected at 1 %.

The control variables (x_i_) are drawn from the empirical literature on health-seeking behaviour in developing countries [11–13, 21]. Age of the household head and number of years of formal education attained by the head of a household are included in the model to capture the effects of human capital on malaria preventive behaviour in the household. Further, household size (number of members 15 years or older) is included to control for family labour supply that may be needed to perform some of the integrated vector management (IVM) related activities–such as bush clearing, waste management or disposal, creating drainage channels. The number of children below 5 years (in the household) is also included to control for vulnerability to malaria, which is likely to influence malaria preventive behaviours in a household.

Further, a dummy variable indicating whether the household head knew the causes and modes of malaria transmission was included to proxy for knowledge of the disease. The effect of household access to public health information was captured in the model using variables on the channels through which they receive important health information—radio, neighbors, television set, print media (news papers, leaf lets, magazines), health workers and school going children. The index takes values from 0–1, corresponding to no information and higher information access, respectively. It was hypothesized that increased access to health related information influences malaria preventive behaviour by increasing knowledge on household health production—thus increasing their likelihood to engage in preventive health care. Furthermore, the proportion of school going children in the household was included to control for the effect of public health awareness in schools on household health behaviour. The study also controls for household per capita income, and it enters the model in logarithmic form. It was hypothesized that, unlike their poor counterparts, households with higher income are likely to adopt more malaria prevention practices, especially the purchased technologies such as window screens, and repellents because they may have liquid capital to purchase them. A recent study [27] shows that relaxing liquidity constraints can increase investment in malaria prevention in households. The effect of neighborhood experience on the adoption of malaria prevention interventions was captured in the model by using the proportion of households in the village that had adopted at least two categories of the integrated vector management (IVM) practices for mosquito control.

Consonant with the social network theory, households located in a village that has greater experience with malaria prevention practices are expected to be more likely to adopt them. Furthermore, we include participation in community groups to control for the influence of community level programmes on adoption of malaria prevention and control technologies. The study further captures household susceptibility to malaria using village level prevalence of the disease; computed as the proportion of households in the village that reported a malaria case prior to the survey.

### Data and summary statistics

The study utilizes a baseline data collected by ICIPE in the year 2013 conducted in its sites for the integrated vector management (IVM) projects located in Nyabondo and Malindi provinces in Kenya. Nyabondo site located in a rural plateau area in Upper Nyakach Division of Kisumu County, western Kenya (coordinates: 00 22′ S; 340 58′ E). Nyabondo project site is situated 30 km on the north eastern part of Lake Victoria, and it lies at an altitude of between 1520 and 1670 m above sea level. Farming including production of crops and livestock are the major economic activities in the area, although households also diversify into non-farm activities particularly brick making. On the other hand, Malindi is located on the shores of the Indian Ocean in Coast Province about 108 km north of Mombasa (coordinates: 30 13′ S; 400 7′ E). Farmer households in Malindi also engage in fishing, and trading for livelihoods. The baseline survey is part of the planned panel data-set to be collected to evaluate the impact of ICIPE’s integrated vector management programme for malaria prevention and control programme in these sub-counties. The survey covered a sample of 2718 households including 1120 drawn from Nyabondo and 1958 from Malindi. The survey questionnaire covered relevant variables including the malaria prevention strategies currently used by the household, household level attributes (sex of the head, household size and wealth), household participation in community group activities, channels through which households get public health information. In this sample, the proportion of female-headed households is about 28.47 %.

**Fig. 1:**
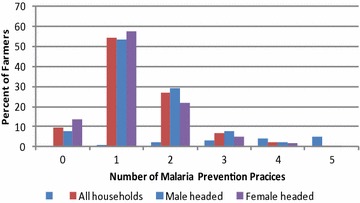
Malaria practices adopted by households

Figure 1 shows distribution of the number of prevention interventions adopted by the different categories of households. Most of the interviewed household in both sites used one practice to prevent malaria. With regard to non-adoption, about 9 % of the households did not use any practice to prevent malaria. The proportion of non-adopters was higher among female-headed households (about 13 %) than their counterparts headed by females (7 %). The graphs further show that adoption decays with the number of technologies.

Table 1 shows the marginal distribution of the prevention technologies used by the surveyed households. As it can be seen from Table 1, ITNs were the most widely used technology (used by about 96 % of the surveyed households), followed by bush clearing (about 71 %), water drainage practices (21 %), proper waste disposal (17 %) and window/door screening (12 %). Relatively small proportions (less than 5 %) of households used indoor residual spray and repellents. Significant differences are observed between male- and female-headed households with respect to adoption of individual malaria preventive practices–with a large proportion of female-headed households using most of the malaria prevention practices compared to their male counterparts.

**Table 1:** Marginal probabilities of adoption of IVM practices

Technology	All households	Male headed HH	Female headed HH	T-statistic	P value
Practices for personal protection					
Insecticide sprays	0.0482	0.0495	0.0450	0.5042	0.6142
	(0.2142)	(0.2169)	(0.2074)		
Use bed nets (ITNs)	0.9595	0.9422	0.9665	2.6115	0.0091
	(0.1971)	(0.2336)	(0.1800)		
Practices to prevent mosquito entry					
Window/door screening	0.1214	0.1221	0.1211	−0.0701	0.9441
	(0.3267)	(0.3276)	(0.3264)		
Purchased repellents	0.0548	0.0386	0.0613	2.5891	0.0097
	(0.2277)	(0.1927)	(0.2400)		
Traditional repellents (plants)	0.0914	0.0567	0.1032	3.2141	0.0014
	(0.2882)	(0.2315)	(0.3043)		
Practices to prevent mosquito breeding					
Water drainage practices	0.2116	0.1967	0.2175	1.2231	0.2215
	(0.4085)	(0.3977)	(0.4127)		
Bush clearing	0.7182	0.6864	0.7309	2.2901	0.0222
	(0.4500)	(0.4643)	(0.4436)		
Proper disposal of containers and trash	0.1755	0.1989	0.11,697	−5.5909	0.0000
	(0.3805)	(0.3993)	(0.3215)		

Summary statistics for selected attributes of the sample are presented in Table 2, for male- and female-headed households. The descriptive statistics show significant differences between the two categories of households with respect to several pre-determined characteristics. For example, the summary statistics reveal that a larger proportion of males had attained some formal education, and had more wealth relative to their female counterparts. About 68 % of the households with male heads were literate compared to only 35 % of the households of female heads. Further, households headed by males reported substantially higher per capita income relative to the female-headed households. However, a larger proportion of female-headed households subscribed to community groups relative to the males.

**Table 2:** Summary statistics of the surveyed households

All households	All households	Male headed	Female headed	T-statistic
Number of technologies adopted	1.525	1.560	1.432	−3.942
	(0.747)	(0.762)	(0.700)	
Age of head of household (years)	43.0503	41.7644	46.2785	6.0622
	(16.6433)	(15.9916)	(17.7826)	
Formal education of head of household (years)	0.5890	0.6830	0.3528	−16.1602
	(0.4921)	(0.4654)	(0.4782)	
No of children under 5 years of age	1.2282	1.3666	0.8806	−10.1608
	(1.1886)	(1.2051)	(1.0707)	
Number of household members >5 years of age	4.7594	4.9836	4.1963	−7.3356
	(2.6944)	(2.7835)	(2.3665)	
Household head subscribes to a community group	0.5878	0.5769	0.6154	1.8296
	(0.4923)	(0.4942)	(0.4868)	
Household access to health related information (index)	0.4164	0.4242	0.3969	−2.3094
	(0.2805)	(0.2845)	(0.2696)	
Neighborhood effects (proportion of adopters in the village)	0.408	0.411	0.399	−1.683
	(0.166)	(0.167)	(0.164)	
Percapita income (Kenya Shillings)	15487.790	17529.130	10261.960	−3.811
	(58193.640)	(66718.110)	(25004.410)	
Study site (1 = Nyabondo, 0 = Malindi)	0.3963	0.3703	0.4615	4.2849
	(0.4892)	(0.4830)	(0.4988)	
Village level prevalence of malaria	0.119	0.117	0.123	2.268
	(0.064)	(0.062)	(0.067)	
Knowledge of malaria transmission (1 = knowledgeable)	0.8961	0.9107	0.8594	−3.5973
	(0.3052)	(0.2852)	(0.3478)	

Standard deviations in parentheses

## Estimation results

Although two separate models were estimated in this study for the determinants of malaria preventive behaviour among male- and female-headed households, a single model for malaria prevention with gender simply as a dummy variable was also estimated as is customary. Column 1 of Table 3 presents estimates of the pooled model derived using a Poisson regression model. The results show that gender of head of a household is significantly associated with the number of malaria practices adopted in the household. The coefficient on gender is positive suggesting that households headed by males in this sample are likely to use more malaria prevention practices compared to their female counterparts. The regression results also show that malaria preventive behaviour is significantly influenced by access to health related information, neighbourhood effects, participation in community activities, knowledge on malaria cause and transmission, formal education of the head of the household, age of household head, and household *per capita* income. In particular, households with more access to health related information use more malaria prevention practices. As noted earlier, access to health information was captured using an index derived from the channels through which a household received information on malaria. This result underscores the importance of use of the integrated approach to disseminate information on malaria prevention and control in the communities. The findings also show that households that participate in community activities were likely to use more malaria prevention practices relative to their non-participating counterparts. Many of the community activities reported such as development meetings and environmental management aimed at addressing development and public health challenges in the communities. Participation in such activities increase public health awareness and may induce behavioural change among participants. Similarly, presence of households using malaria prevention practices in the neighbourhood induced increased uptake of malaria prevention practices among household, suggesting that learning from other members in the community plays a significant role in technology adoption. Furthermore, households with heads who are knowledgeable about the cause and transmission of malaria used more practices for the disease prevention and control. The results also show that uptake of malaria prevention practices increased before decreasing with age of the head of a household. Furthermore, the coefficient for the location (sub-county) is negative and significant; suggesting that adoption of malaria prevention practices is higher in Malindi relative to Nyabondo. As earlier mentioned, Malindi site is located close to a relatively peri-urban area and is a major tourist hub of Kenya, which makes it a higher target for public health programmes relative to other areas such as Nyabondo; indeed, several public health interventions including malaria control programmes have been implemented by the ministry of health in this site.

**Table 3:** Determinants of adoption of malaria prevention in rural Kenya

Characteristic	Pooled model (n = 2718)	Male headed (n = 1940)	Female headed (n = 778)
Age of head of household (years)	0.0100***	0.00539*	0.0190***
	(0.00268)	(0.00282)	(0.00597)
Square of age of head of household (years)	−0.000121***	−6.46e-05**	−0.000229***
	(2.95e-05)	(3.15e-05)	(6.51e-05)
Village level prevalence of malaria	0.0897	0.0662	0.162
	(0.0662)	(0.0774)	(0.125)
Years of formal education of head of household	0.00510*	0.00596*	0.00133
	(0.00292)	(0.00327)	(0.00627)
Proportion of children in school	0.0105	0.0181	−0.00669
	(0.0200)	(0.0233)	(0.0380)
No of children under 5 years of age	0.00950	0.0172	0.00234
	(0.0105)	(0.0118)	(0.0236)
Household size (no of household members)	0.00281	0.00221	0.000223
	(0.00466)	(0.00527)	(0.0105)
Household head participates community activities (1 = yes)	0.140***	0.115***	0.191***
	(0.0226)	(0.0264)	(0.0433)
Household access to health related information (index)	0.338***	0.357***	0.265***
	(0.0474)	(0.0565)	(0.0888)
Neighborhood effects (proportion of adopters in the village	0.839***	0.802***	0.961***
	(0.0463)	(0.0510)	(0.108)
Dummy for knowledge of malaria cause and transmission	0.148***	0.0984**	0.244***
	(0.0387)	(0.0445)	(0.0789)
Log of household percapita income (Kenya shillings)	0.0162*	0.0213**	0.00514
	(0.00958)	(0.0107)	(0.0223)
Dummy for missing income values	0.228***	0.319***	0.0280
	(0.0869)	(0.0978)	(0.196)
Study site (1 = Nyabondo, 0 = Malindi)	−0.215***	−0.202***	−0.229***
	(0.0358)	(0.0403)	(0.0766)
Gender of head of household (1 = male; 0 = female)	0.0676**		
	(0.0266)		
Constant	−0.573***	−0.444***	−0.678***
	(0.109)	(0.120)	(0.244)

Standard errors in parentheses

*** p < 0.01, ** p < 0.05, * p < 0.1

The above pooled model does not allow us to ascertain if the determinants of malaria preventive behaviour differ between male- and female-headed households. Drivers may differ for a number of reasons. First, there is evidence that female-headed households in developing countries are, on average, financially worse-off (see; [23, 28]) than male-headed households and thus have more binding constraints on investment in purchased technologies given limited access to financial resources. Second, female-headed households may be less likely to adopt practices for malaria prevention because of limited access to information. As further indicated by the summary statistics (Table 2), there are significant differences between male and female households with respect to important socio-economic characteristics, particularly access to health related information, formal education and knowledge of malaria transmission. More male heads were literate, and were knowledgeable about malaria transmission than females. Similarly male headed households had more access to health related information than their female counterparts.

The coefficient estimates and standard errors for male and female-headed households are displayed in columns 2 and 3 of Table 3. Our findings show some differences between the determinants of adoption in male and headed households. For example, the coefficients on education and *per capita* income, are significant in male-headed households but not in their female-headed counterparts. In particular, households headed by males who attained more years of formal education are likely to adopt more malaria prevention practices. Similarly, higher *per capita* income is likely to increase the number malaria prevention practices used by a household. This especially true for purchased technologies such as repellents and insecticide sprays.

## Conclusions

This paper examines the role of gender in malaria preventive behaviour in Kenya using a survey of 2718 households from Malindi and Nyabondo study sites. The study focused on malaria preventive behaviour because malaria prevalence remains high in Kenya and adoption of technologies to prevent the disease remain low, yet they are widely available in the country. While a number of studies have analysed the determinants of malaria prevention in many developing countries, empirical work on its determinants in Kenya is scarce. The study contributes to existing literature by exploring the role of gender of the household head on adoption of malaria prevention practices in households. The results show that male-headed households adopted more practices for malaria prevention than female-headed households. This study further delves into the determinants of adoption of malaria prevention practices for female- and male-headed households to determine if the drivers of adoption differ between the households. The study findings show that access to health information, residing in villages with higher prevalence of malaria preventive behaviour, formal education, wealth (livestock value), and knowledge on malaria cause and transmission increase the number of malaria preventive practices adopted in a male-headed household. For their female-headed counterparts, we find access to health information, residing in villages with higher experience in malaria prevention, knowledge on malaria cause and transmission significant but formal education of and asset endowment insignificant. These findings generally suggest that universal policy tools may be effective in promoting uptake of integrated malaria prevention practice, for female- and male-headed households. In particular, policies which increase public health information, knowledge about the disease (causes, and control), and interaction with adopting neighbours, will have the most pronounced effect on increasing adoption of malaria prevention practices in both male- and female-headed households. In particular, public and private health services providers can reduce the gender gap in access to information on malaria prevention and control by targeting female social networks—to facilitate inclusive dissemination of information and malaria prevention practices.

Although education and *per capita* income were not significant in the model for female-headed households, it is important to implement policy interventions that facilitate inclusive access to formal education and income generation can increase adoption of integrated malaria prevention practices by women. For instance, the Government and development partners in the health sector can improve literacy and numeracy skills of women by integrating female adult literacy in the interventions for malaria prevention and control. Similarly, promotion of non-farm income generation among women can improve the purchasing power of households, and thus likely to increase adoption of purchased malaria prevention practices among female-headed households.
